# microRNA-126 Is a Tumor Suppressor of Granulosa Cell Tumor Mediated by Its Host Gene EGFL7

**DOI:** 10.3389/fonc.2019.00486

**Published:** 2019-06-11

**Authors:** Jiajie Tu, Hoi-Hung Cheung, Gang Lu, Clement Leung-Kwok Chan, Zijiang Chen, Wai-Yee Chan

**Affiliations:** ^1^CUHK-SDU Joint Laboratory on Reproductive Genetics, Faculty of Medicine, School of Biomedical Sciences, The Chinese University of Hong Kong, Hong Kong, China; ^2^Institute of Clinical Pharmacology, Anhui Medical University, Hefei, China; ^3^Women's Health and Reproductive Medicine Centre, Hong Kong, China; ^4^National Research Center for Assisted Reproductive Technology and Reproductive Genetics, Jinan, China; ^5^Center for Reproductive Medicine, Shandong University, Jinan, China

**Keywords:** miR-126, GCT, EGFL7, AKT pathway, tumoriogenesis

## Abstract

MicroRNAs (miRNAs) are small noncoding RNAs that regulate gene expression at a post-transcriptional level. We examined the role of miR-126 in granulosa cell tumor (GCT) of the ovaries. In tissues from malignant GCT patients miR-126 expression was repressed. We showed that miR-126 could inhibit proliferation, migration, hormone production and promote apoptosis of cancerous granulosa cells (GCs) *in vitro*. The role of miR-126 as “tumor suppressor” was confirmed by using a tumor formation model *in vivo*. By RNA-seq, immunohistochemical staining (IHC), Western blot and luciferase reporter assay, we identified and confirmed EGFL7 as a direct functional target of miR-126 in cancer GCs. Furthermore, we found that the AKT signaling pathway was associated with miR-126 and EGFL7 in cancer GCs. Taken together, our results demonstrate a function of miR-126 in the suppression of GCT development via the regulation of EGFL7.

## Introduction

Ovarian cancer is one of the most common types of cancer in women. It has a poor general prognosis of survival due to the generally late diagnosis (1). The three most abundant subtypes of ovarian cancer are ovarian epithelial cancer, germ cell cancer and granulosa cell (GC) tumors (GCT) (2). GCT accounts for about 5% of all ovarian cancers with a high rate of recurrence but the pathogenesis of this form is not well understood. Therefore, it is essential for prognosis and therapy of GCT patients to understand the underlying pathogenic mechanisms of GCT.

Target gene expression has been shown to be affected through a direct binding of micro RNAs (miRNAs), a group of small non-coding RNAs, to the 3′-untranslated region of their target genes (3). The essential regulatory role of miRNAs has been proven in a variety of cancers (4). miRNAs are active in drug resistance during chemotherapy and their involvement in the development of epithelial ovarian cancer (EOC) has been deduced from a number of gene profiling studies (5).

The precise target genes of miRNAs in GCT tumorigenesis have not yet been identified. One candidate, Epidermal growth factor-like domain-containing protein 7 (EGFL7) has been proven to be a critical oncogene in various types of cancer (6–10). Notably, EGFL7 is highly expressed in patients with EOC and its expression has been correlated with a poor prognosis for these patients (11). In addition, EGFL7 also serves as a potential predictive marker of chemotherapy for cervical cancer (12). The role of EGFL7 in GCT, however, is largely unknown. Interestingly, a microRNA, miR-126, embeds in the genomic region of EGFL7. A study reports methylation-associated silencing of miR-126 and its host gene EGFL7 in pleural mesothelioma (13), suggesting an association between EGFL7 and miR-126 in cancer. Due to the similarities between mesothelial lineage tumor and GCT, we try to illuminate the role of miR-126 and EGFL7 in GCT. In our present study, we show that miR-126 constrains the tumorigenesis of GCT via directly targeting EGFL7 and consequently suppresses the phosphatidylinositol 3-kinase/ATK (PI3K/AKT) pathway. Our data suggest a critical, physiological role for miR-126 in the GCT oncology. Significantly, it may be utilized as a prognostic marker or a therapeutic target for GCT treatment.

## Materials and Methods

### Fluorescence *in situ* Hybridization (Fish)

A cohort of 60 primary ovarian GCT (44 samples of benign and 6 samples of malignant tumor samples. Ten cases of ovarian cyst) was obtained from US Biomax. Detection probes for miR-126 (miRCURY LNA miRNA) were purchased from Exiqon (Denmark). The assays were performed according to Exiqon's standard protocol of miRNA FISH. To avoid experimental bias the samples were blinded before analysis.

### Cell Culture

Two human GCT lines, the adult GCT cell line KGN (14), the juvenile GCT cell line Cov434 (15) and normal human GC line SVOG (16) were kindly provided by Prof Zijiang Chen (Shandong University). KGN cells were cultured in DMEM/F12 media (Gibco) with 10% FBS and 1% penicillin/streptomycin. Cov434 cells were cultured in DMEM media (Gibco) with 10% FBS, 1% penicillin/streptomycin and 1% NEAA. As controls, HEK293T cells were purchased from Invitrogen and cultured in DMEM media (Gibco) with 10% fetal bovine serum and 1% penicillin/streptomycin. A 37°C/5% CO_2_ humidified incubator was used for cell culture.

#### miRNA Target Prediction

miR-126 target prediction was performed by using TargetScan (http://www.targetscan.org/). EGFL7 was predicted as a direct target of miR-126.

### miRNA Mimics/Inhibitor

miR-126 mimics/inhibitor and scrambled control were obtained from GenePharma. A lipofectamine transfection reagent (RNAiMAX, Life Technologies) was used for transfection of miR-126 mimics/inhibitor.

### Lentivirus and Retrovirus

EGFL7 short hairpin (sh)RNAs were designed using the webpage http://sirna.wi.mit.edu/home.php. The vector pLVTHM (Addgene No. 12247) was used for shRNA cloning. Lentivirus packaging was carried out according to the manufacturers' protocols. Green fluorescent protein (GFP) sorting was used to isolate successfully transfected cells (17). MDH1-PGK-EGFP plasmid is a retroviral construct for miR-126 expression (Addgene No. 11375). Quantitative RT-PCR and western blot were used to validate knockdown effect.

### Total and Small RNA Extraction, Reverse Transcription and qPCR

Total RNA was extracted with Trizol reagent (Invitrogen) according to standard protocol. Concentration and quality of all RNA samples were evaluated by Nanodrop 2000 (Thermo). MasterMix kit (Takara) and TaqMan reverse transcription kit (Life Technology, USA) were used for mRNA and miRNA reverse transcription. Universal SYBR Green Master mix (Applied Biosystems) and TaqMan specific microRNA probe (Life technology) were used for qPCR assays. All qPCR were performed by StepOnePlus real-time PCR system (Applied Biosystems). GAPDH and U6 snoRNA were used for normalization of mRNA and miRNA.

### Proliferation

Cell-counting kit-8 was used for a quantification of proliferation.

### Apoptosis

Alexa Fluor®488 annexin V/Dead Cell Apoptosis Kit (Invitrogen, CA, USA) was used for apoptosis evaluation according to the manufacturer's guidelines.

### Invasion and Migration

Transwell plates with inserts were used for invasion and migration assays (Costar) Matrigel (BD) diluted to 0.1 mg/ml with DMEM media was used to coat the upper chamber for the invasion assay. KGN cells (2 × 10^4^ cell per chamber) were seeded into the upper chamber with FBS-free medium and 10% FBS medium was added into the lower wells. After 24 h, the transwell chambers were washed and stained with 1% crystal violet.

### ELISA

Supernatants of KGN cells were collected after different treatments. Human Estron competitive ELISA kit (Invitrogen) and Estradiol human ELISA kit (Thermofisher) were used for Estrone and Estrodial detection.

### Immunohistochemical Staining (IHC)

Paraffin embedded tissue slides that obtained from KGN cells-formed tumors were blocked in 5% goat serum for half hour, then incubated in primary anti-PCNA (Santa Cruz), P-Akt (Cell Signaling) and EGFL7 (Santa Cruz) antibodies at 4°C for 12 h (18).

### RNA-Sequencing

The total RNA was collected from KGN cells that transfected with miR-126 mimics and negative control. The RNA quality was measured by NanoDrop2000 spectrophotometer. RNA-seq and subsequent transcriptome analysis were carried out by GROKEN Bioscience (China) according to standard protocol.

### Western Blot

Cells were lysed in SDS buffer (100 mM Tris-Cl (pH 6.8), 4% SDS (sodium dodecyl sulfate), 0.2% bromophenol blue, 20% glycerol, 200 mM β-mercaptoethanol). BCA assay kit (Thermofisher) was used for protein measurement. The primary antibodies used were listed as follows: EGFL7 (Santa Cruz), PI3K (Abcam), AKT (Abcam), S6K (Cell signaling), mTOR (Abcam), p-AKT (Abcam), FOXO1 (Abcam). GAPDH (Cell signaling) and β-ACTIN (Santa Cruz) were used as loading controls.

### Luciferase Reporter Assay

HEK293T cells were co-transfected with miR-126 mimics, pmirGLO vector (Promega) tagged with EGFL7 3′UTR by using Lipofectamine 2000 (Invitrogen). The empty pmirGLO plasmid was used as negative control. Dual-Luciferase Reporter Assay System (Promega) was used for Firefly/Renilla luciferase activities.

### Tumor Formation *in vivo* Model

The related *in vivo* experiments were approved by the Animal Research Committee at the LASEC, Chinese University of Hong Kong. All nude mice used in this experiment were obtained from LASEC. For the ectopic tumor formation model (19), Control KGN cells and miR-126 ovexpressing KGN cells were injected subcutaneously (5 × 10^6^ cells/mice) in 4–6 weeks female nude mice (8 mice for each group). All mice were sacrificed after 4 weeks. Then cancerous GC-formed tumor were isolated for following analysis.

### Statistics

Prism (GraphPad) was used for statistical tests. Unpaired, two-tailed Student's *t*-tests was used for parametric results. One-way ANOVA with Tukey's *post-hoc* tests was used to further examine pairwise differences. Two-sided Mann-Whitney test was performed for non-parametric results. A level of *P* < 0.05, was used to designate significant differences. ^*^, ^**^, and ^***^ indicate *P* < 0.05, *P* < 0.01, and *P* < 0.001, respectively.

## Results

### miR-126 Represses Cancerous GCs Functions *in vitro*

**Figure 1:**
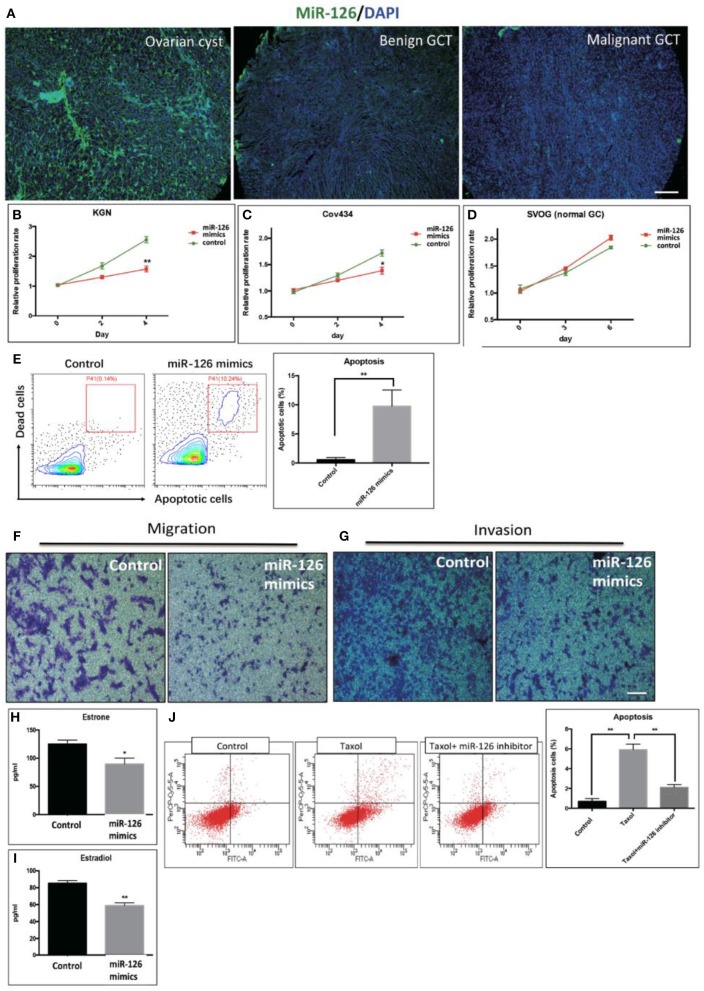
The effect of miR-126 overexpression on cancerous GCs *in vitro*. **(A)** Fluorescence *in situ* hybridization (FISH) studies detected a weaker staining of miR-126 in malignant GCT tissues than either benign GCT tissues or samples of ovarian cysts (scale bar = 200 μm). **(B,C)** Proliferation of two GCT cell lines was induced by miR-126. **(D)** miR-126 slightly promoted proliferation of normal GCs. **(E)** miR-126 induced apoptosis of KGN cells. Control: KGN cells +NC; miR-126 mimics: KGN cells +mimics. **(F,G)** miR-126 overexpression inhibited cancerous GC migration and invasion (scale bar = 100 μm). **(H,I)** miR-126 repressed productions of estrone and estradiol from KGN cells. **(J)** miR-126 promoted Taxol-induced cell death in cancerous GCs. Control: KGN cells+NC; Taxol: KGN cells+NC+Taxol; Taxol+miR-126 inhibitors: KGN cells+Taxol+inhibitors; Each bar in the figure represents the mean ± SEM of triplicates. *P < 0.05, **P < 0.01.

The expression level of miR-126 in clinical specimens obtained from a biobank of a GCT cohort was analyzed using fluorescence *in situ* hybridization (FISH). The endogenous level of miR-126 in was examined in 60 GCT patients. Our results showed that miR-126 expression was significantly lower in both malignant and benign GCT tissues than in non-cancerous ovarian cyst material (Figure 1A). To investigate the effect of miR-126 on the oncogenesis of cancerous GCs *in vitro*, miRNA mimics and inhibitors were transfected into two GCT patient-derived cancer cell lines, KGN cells (derived from an adult GCT patient) and COV434 cells (derived from a juvenile GCT patient) (Supplementary Figure 1). Proliferation of these two cancerous GCs was significantly repressed (Figures 1B,C) while, simultaneously, apoptosis was induced by miR-126 mimics in cancerous GCs (Figure 1E). By contrast, miR-126 didn't affect proliferation of a normal GC line, SVOG (14) (Figure 1D), suggesting that the proliferation-inhibitory effect of miR-126 was more evident in cancerous GCs. Cell migration and invasion are the two characteristics of metastasis. Using *in vitro* transwell assays, our results showed that miR-126 could inhibit both migration and invasion of KGN cells (Figures 1F,G). In addition, estrone and estradiol are highly secreted by GC in GCT patients. The abnormal production of estrone and estradiol promotes GCT development. ELISA results indicated that miR-126 could repress secretion of estrone and estradiol from KGN cells (Figures 1H,I). Chemoresistance of KGN cells to anti-cancer drugs was measured by apoptosis assay after treating cells with Taxol, a common anti-cancer drug. The results revealed that the Taxol-induced cells death is, at least partially, miR-126 dependent (Figure 1J). In addition, similar *in vitro* results were obtained using Cov434 cells (Supplementary Figure 1). These *in vitro* experiments suggest a tumor suppressor role for miR-126 in GCT, which might account for its low expression in the malignant form of GCT.

### The Effect of miR-126 on Cancerous GCs at Transcriptome Level

**Figure 2:**
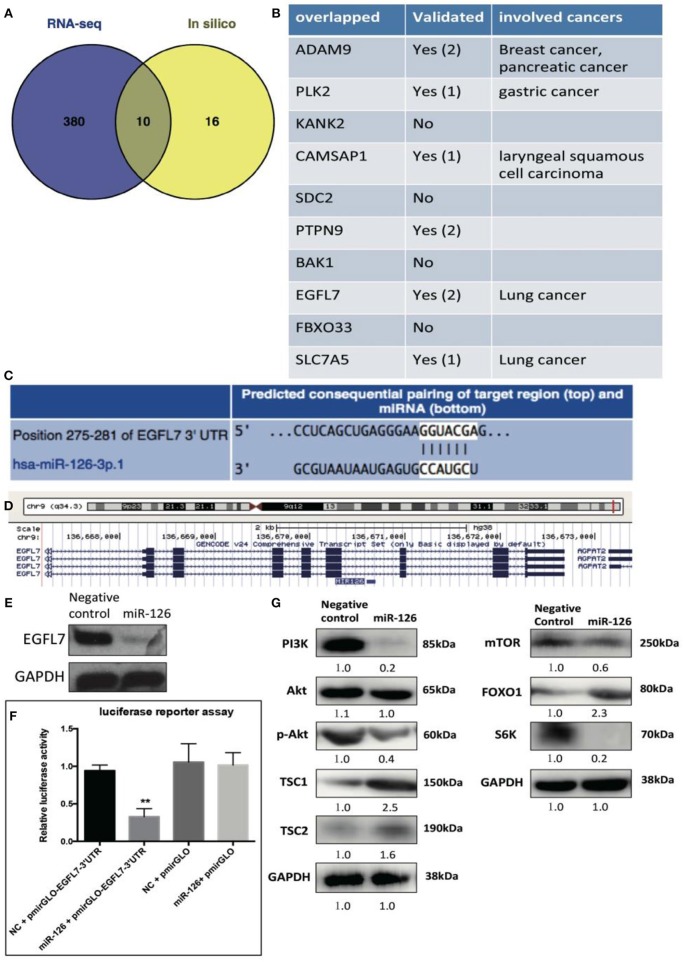
The effect of miR-126 on cancerous GCs at transcriptome level. **(A)** Venn diagram compares all miR-126 repressed genes in cancerous GCs by RNA-seq and miR-126 predicted targets by bioinformatics prediction. **(B)** The related information of all 10 overlapped genes from Venn comparison. **(C)** The predicted binding sites of miR-126 and EGFL7 from TargetScan website (http://www.targetscan.org/). **(D)** MiR-126 embeds in the third intron of EGFL7. **(E)** MiR-126 represses EGFL7 expression in cancerous GCs. **(F)** Luciferase reporter assay shows the directly binding between miR-126 and EGFL7 3'UTR in cancerous GCs. **(G)** MiR-126 regulates AKT pathway in cancerous GCs. Each bar in the figure represents the mean ± SEM of triplicates. **P < 0.01.

We reasoned that the “tumor suppressor” role of miR-126 in cancerous GCs might be coupled with gene(s) or signaling pathway(s) that change the cell behavior. To unlock the underlying molecular mechanism at the transcriptional level, RNA-seq was performed to compare the difference in gene expression between control and miR-126 overexpressing cancerous GCs (Supplementary Figure 2). Results from KEGG pathway analysis revealed that 11 of the top 20 miR-126-associated pathways were cancer-associated (Supplementary Figure 2). Some previously reported GCT-related pathways, such as the estrogen signaling and the PI3K-AKT pathways, were also found in the list (Supplementary Figure 2). Next, we attempted to identify the key functional targets of miR-126 in cancerous GCs. By comparing all down-regulated genes from the RNA-seq analysis and with the help of software prediction, 10 candidate genes were identified (Figure 2A). Interestingly, in line with literal reports, five of the candidate genes have been proven of playing significant functions in different types of cancer (Figure 2B). To confirm the putative functional targets of miR-126 specifically in cancerous GCs, shRNAs were employed to knock down each individual gene among the 10 candidates (Supplementary Figure 3). After validation of the knockdown efficiency in KGN cells, CCK-8 assay was performed to examine the knockdown effect on the proliferation of cancerous GCs (Supplementary Figure 3). While the knockdown of 9 genes had negligible effects on KGN cell proliferation it was significantly reduced upon EGFL7 knockdown. This observation was consistent with the result of miR-126 overexpression that led to proliferation arrest in cancerous GCs (Figures 1B,C). Interestingly, genomic analysis indicated that EGFL7 was the target gene that contained the miR-126 binding site (Figure 2D). We also performed Western blotting to confirm the RNA-seq results, concluding that miR-126 could repress EGFL7 expression in cancerous GCs at the protein level (Figure 2E). To determine whether miR-126 directly binds to the EGFL7 3′UTR and suppresses its translation (Figure 2C), a luciferase reporter assay demonstrated that miR-126 binding to the EGFL7 3′UTR could indeed decrease the reporter activity (Figure 2F). Additionally, we also demonstrated in the PI3K-AKT pathway, a miR-126 associated, oncogenic pathway in GCT, was altered following miR-126 ovexpression (Figure 2G). A series of essential proteins in PI3K-AKT pathway, including PI3K, p-AKT, TSC1, TSC2, mTOR, FOXO1, and S6K, were modulated by miR-126 (Figure 2G). In summary, through transcriptional analyses and biochemical and cellular assays, we established a functional link between EGFL7 and miR-126 in the regulation of GC tumorigenesis, potentially through the PI3K-AKT pathway.

### miR-126 Inhibits GCT Formation *in vivo*

**Figure 3:**
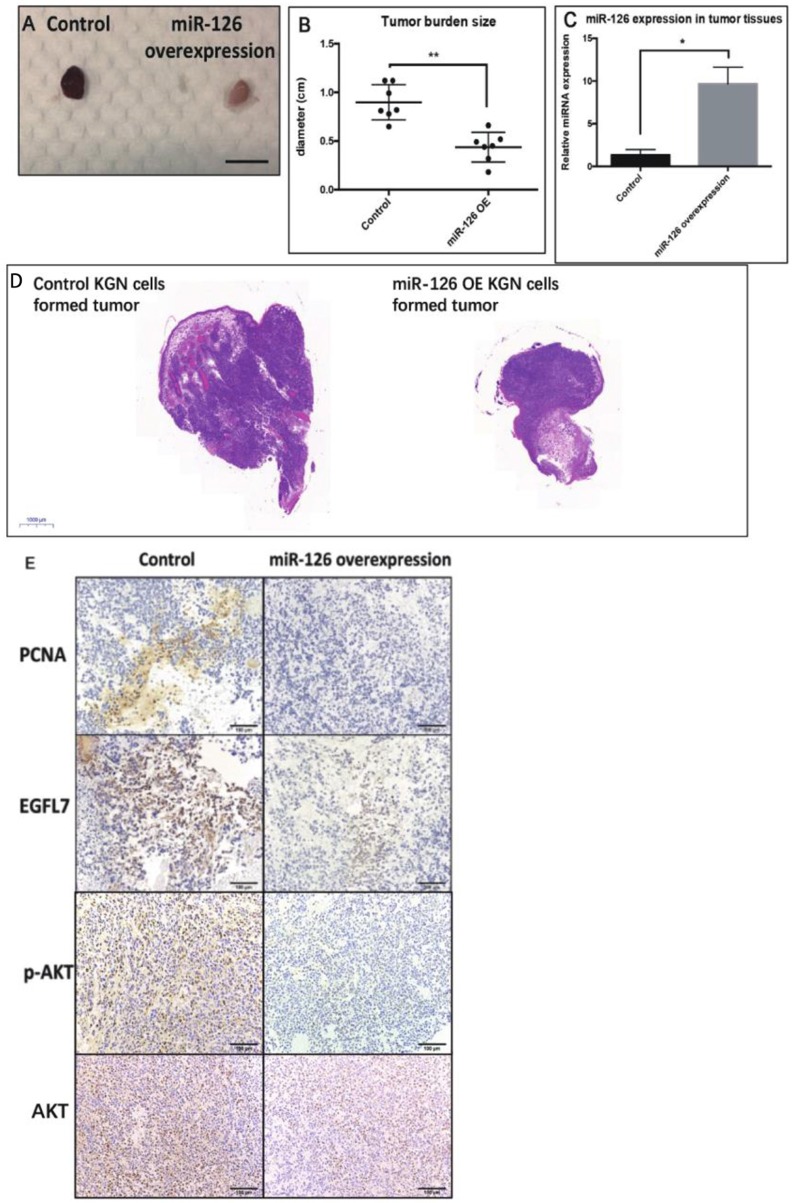
The “tumor-suppressor” role of miR-126 on GCT *in vivo*. **(A,B)** The size of miR-126 overexpressing KGN cells-formed tumor was generally smaller than that of control KGN cells (scale bar = 150 mm). **(C)** miR-126 highly expressed in miR-126 overexpressing KGN cells-formed tumor tissues. **(D)** The H&E staining of control and miR-126 overexpressing KGN-formed tumors. **(E)** The effects of miR-126 on proliferation marker PCNA, EGFL7 and components of AKT pathways (p-AKT) in control KGN cells miR-126 overexpressing KGN cells-formed tumor. Each bar in the figure represents the mean ± SEM of triplicates. Eight mice were used in each group. *P < 0.05, **P < 0.01.

To investigate whether miR-126 inhibits GCT growth *in vivo*, an ectopic tumor formation mice model was used. A KGN cell line that stably over-expressed miR-126 was generated (Supplementary Figure 1) and control or miR-126 overexpressing KGN cells were subcutaneously injected to the flank back of nude mice. After 4 weeks, the cancerous GCs formed solid tumors. The size of tumors formed by miR-126 overexpressing KGN cells was significantly smaller than that of tumors formed by untransfected control cells (Figures 3A,B,D). Quantitative PCR results confirmed that miR-126 expression was significantly induced in miR-126 overexpressing KGN cells-formed tumor (Figure 3C). Immunohistochemical analysis revealed that PCNA, a proliferation marker, was significantly decreased in miR-126 overexpressing tumor cells (Figure 3E). Consistently, we also observed suppression of EGFL7 in these tumor tissues (Figure 3E). In addition, we also investigated the expression of p-AKT, the critical node of the AKT pathway. Result from IHC showed that this protein was significantly downregulated in miR-126 overexpressing KGN tumors (total AKT didn't change), indicating that AKT pathway was altered by miR-126 *in vivo*. To summarize, our results demonstrated a “tumor suppressor” role for miR-126 in GCT. EGFL7 appeared as a relevant target of miR-126 in mediating the effect of the miRNA, in part by changing the AKT pathway.

### Stable Knockdown of EGFL7 Mimics miR-126 Overexpression-Mediated Effects in Cancerous GCs

**Figure 4:**
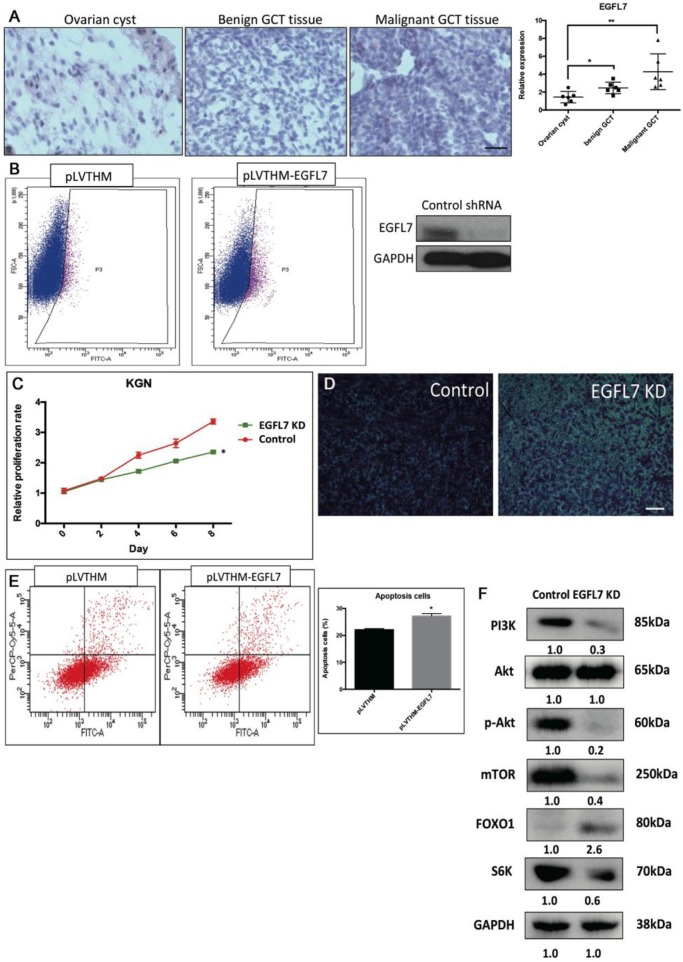
The oncogenic effect of EGFL7 in cancerous GCs. **(A)** EGFL7 highly expresses in the malignant GCT tissue (*n* = 6). **(B)** The transfected cancerous GCs were isolated by cell cytometry and the knockdown effect of EGFP-labeled shRNA on endogenous EGFL7 in cancerous GCs was validated by western blotting. **(C)** The effect of EGFL7 knockdown on proliferation of cancerous GCs. **(D)** The effect of EGFL7 knockdown on migration of cancerous GCs. **(E)** The effect of EGFL7 knockdown on apoptosis of cancerous GCs. **(F)** The effect of EGFL7 knockdown on AKT pathway in cancerous GCs. Each bar in the figure represents the mean ± SEM of triplicates. Scale bar = 50 μm, *P < 0.05, **P<0.01.

We found that a stable knockdown of EGFL7 could inhibit proliferation of cancerous GCs (Supplementary Figure 3). This anti-mitotic effect was similar to that of miR-126 overexpression (Figure 4B). To investigate whether EGFL7 is a critical target for mediating miR-126 function in GC tumorigenesis, we examined EGFL7 expression in GCT tissues. Compared to ovarian cyst and benign GCT tissues, EGFL7 was highly expressed in malignant GCT tissues (Figure 4A), which is diametrically opposed to the expression of miR-126 in GCT tissues. To demonstrate a role of EGFL7, a lentivirus-based stable knockdown of EGFL7 was performed. Following the sorting of positively transduced cells (Figure 4B), the expression of EGFL7 was stably decreased, as validated by Western blot (Figure 4B). In these EGFL7 deficient KGN cells, proliferation and migration were found to be decreased (Figures 4C,D). In addition, there were more apoptotic cells in the EGFL7 stable knockdown KGN cells (Figure 4E). Similar as in miR-126 overexpressing cancerous GC, the AKT pathway was also repressed in EGFL7 KD cancerous GC (Figure 4F), further confirming that the effect of the miR-126/EGFL7 regulatory axis on cancerous GC is mediated by AKT pathway. Taken together, these results demonstrated that EGFL7 depletion imitated the effect of miR-126 overexpression indicating EGFL7 as a functional target of miR-126 in cancerous GCs.

## Discussion

Although there is an emerging comprehension of the mechanism of GCT pathogenesis further details of pathogenesis need to be established (20), before a future implementation of these new insights into diagnosis or clinical treatment of GCT can be envisaged. Therefore, the challenging investigation of essential oncogene/tumor-suppressor genes in transcription and metabolic pathways in GCT is essential for developing an alternative, targeted therapy, especially in advanced-stage cancer. In particular because the mechanisms underlying oncogenesis are still controversial. Due to the rare occurrence of GCT, there are still no specific and robust clinical biomarkers for GCT with the potential exception of the *FOXL2* mutation that has been suggested as biomarker in adult GCT (21). However, this genetic marker is not suitable for mutation-negative adult GCT or juvenile GCT. Therefore, we investigated the possibility of using miRNAs as novel markers for GCT.

The role of miR-126 has been independently elucidated in leukemia, renal and gastric cancer and various other forms (22–33). However, in ovarian GCT the question of a role of miR-126 has not yet been addressed. In our study, we found that miR-126 expression was significantly down-regulated in both benign and malignant GCT tissues when compared to ovarian cyst tissues indicating a potential tumor-suppressor role. The tumor-suppressor role of miR-126 was further validated in both *in vitro* (proliferation, migration/invasion, apoptosis, estrone/estradiol production and drug-resistance) and *in vivo* (tumor formation) assays. In addition, using RNA-seq based transcriptome profiling we compared down-regulated genes with *in silico* target prediction and identified 10 candidate genes. Interestingly, results from literature research showed that 6 of the genes (*ADAM9, PLK2, CAMSAP1, PTPN9, EGFL7* and *SLC7A5*) have been demonstrated as the functional targets of miR-126 (23, 34–42), suggesting the relevance of our analysis. To identify the GCT-relevant target of miR-126, we screened all the 10 candidate genes in KGN cells by RNAi. Using cell growth as an indicator for tumorigenesis (by CCK assay), only *EGFL7* knockdown was revealed to slow down cell proliferation.

A previous study has reported methylation-associated silencing of miR-126 and its target gene *EGFL7* in pleural mesothelioma (13) suggesting a close association between *EGFL7* and miR-126 in cancers. In the present study, *EGFL7* is shown as a functional target of miR-126. In line with this, knockdown of *EGFL7* in cancerous GCs showed similar phenotype to a miR-126 overexpression, suggesting that miR-126 suppresses the development of GCT, at least partially, via down-regulating *EGFL7*.

The AKT signaling pathways have been recognized as a critical in GCT oncogenesis (43, 44). Interestingly, it has been demonstrated that EGFL7 could activate both AKT pathways in glioma and gastric cancer (6, 10). In our study, the AKT pathway was also activated by miR-126 in cancerous GCs, which is in line with other reports. Therefore, we suggest that miR-126-EGFL7-AKT regulatory axis may play an important role in driving GCT development. In the future, the specific regulation and function of this regulatory axis in GCT by miR-126 will be delineated in more details.

Taken together, our current report highlights a hitherto unnoticed role of miR-126 in GCTs, and implies a mechanism by which the miR-126-EGFL7 axis and the AKT pathway are activated in GCT. This finding may indicate that the association of miR-127 and EGFL7 could be useful as a potential biomarker and ultimately, as a therapeutical target for GCT.

## Ethics Statement

This study was carried out in accordance with the recommendations of Animal Research Committee at the LASEC, Chinese University of Hong Kong. The protocol was approved by Department of Health (Hong Kong).

## Author Contributions

JT and W-YC conceived and designed all the experiments. JT performed the experiments. JT, H-HC, GL, CC, ZC, and W-YC drafted and revised the article. All authors read and approved the final version.

### Conflict of Interest Statement

The authors declare that the research was conducted in the absence of any commercial or financial relationships that could be construed as a potential conflict of interest.

## References

[1] Hunn J Rodriguez GC. Ovarian cancer : etiology, risk factors, and epidemiology. Clin Obstet Gynecol. (2012) 55:3–23. 10.1097/GRF.0b013e31824b4611 22343225

[2] Coleman RL Monk BJ Sood AK Herzog TJ. Latest research and treatment of advanced-stage epithelial ovarian cancer. Nat Rev Clin Oncol. (2013) 10:211–24. 10.1038/nrclinonc.2013.5 23381004 PMC3786558

[3] Ha M Kim VN. Regulation of microRNA biogenesis. Nat Rev Mol Cell Biol. (2014) 15:509–24. 10.1038/nrm3838 25027649

[4] Hayes J Peruzzi PP Lawler S. MicroRNAs in cancer: biomarkers, functions and therapy. Trends Mol Med. (2014) 20:460–9. 10.1016/j.molmed.2014.06.005 25027972

[5] Zhang S Lu Z Unruh AK Ivan C Baggerly KA Calin GA. Clinically relevant microRNAs in ovarian cancer. Mol Cancer Res. (2015) 13:393–401. 10.1158/1541-7786.MCR-14-0424 25304686 PMC4369176

[6] Wang FY Kang CS Wang-Gou SY Huang CH Feng CY Li XJ. EGFL7 is an intercellular EGFR signal messenger that plays an oncogenic role in glioma. Cancer Lett. (2017) 384:9–18. 10.1016/j.canlet.2016.10.009 27725228

[7] Huang C Yuan X Wan Y Liu F Chen X Zhan X. VE-statin/Egfl7 expression in malignant glioma and its relevant molecular network. Int J Clin Exp Pathol. (2014) 7:1022–1031. 24696719 PMC3971305

[8] Hansen TF Andersen RF Olsen DA Sørensen FB Jakobsen A. Prognostic importance of circulating epidermal growth factor-like domain 7 in patients with metastatic colorectal cancer treated with chemotherapy and bevacizumab. Sci Rep. (2017) 7:1–9. 10.1038/s41598-017-02538-x 28539619 PMC5443778

[9] Papaioannou D Shen C Nicolet D McNeil B Bill M Karunasiri M. Prognostic and biological significance of the proangiogenic factor EGFL7 in acute myeloid leukemia. Proc Natl Acad Sci USA. (2017) 114:E4641–7. 10.1073/pnas.1703142114 28533390 PMC5468639

[10] Luo BH Xiong F Wang JP Li JH Zhong M Liu QL. Epidermal Growth Factor-Like domain-containing protein 7 (EGFL7) enhances EGF receptor-AKT signaling, epithelial-mesenchymal transition, and metastasis of gastric cancer cells. PLoS ONE. (2014) 9: e99922. 10.1371/journal.pone.0099922 24945379 PMC4063792

[11] Oh J Park SH Lee TS Oh HK Choi JH Choi YS. High expression of epidermal growth factor-like domain 7 is correlated with poor differentiation and poor prognosis in patients with epithelial ovarian cancer. J Gynecol Oncol. (2014) 25:334–41. 10.3802/jgo.2014.25.4.334 25142627 PMC4195305

[12] Yamauchi M Fukuda T Wada T Kawanishi M Imai K Tasaka R. Expression of epidermal growth factor-like domain 7 may be a predictive marker of the effect of neoadjuvant chemotherapy for locally advanced uterine cervical cancer. Oncol Lett. (2016) 12:5183–9. 10.3892/ol.2016.5318 28105226 PMC5228482

[13] Andersen M Trapani D Ravn J Sørensen JB Andersen CB Grauslund M. Methylation-associated silencing of microrna-126 and its host gene egfl7 in malignant pleural mesothelioma. Anticancer Res. (2015) 35:6223–9. 26504055

[14] Dou YD Zhao H Huang T Zhao SG Liu XM Yu XC. STMN1 promotes progesterone production Via StAR Up-regulation in mouse granulosa cells. Sci Rep. (2016) 6:1–10. 10.1038/srep26691 27270953 PMC4897624

[15] Zhao S Xu H Cui Y Wang W Qin Y You L. Metabolic actions of insulin in ovarian granulosa cells were unaffected by hyperandrogenism. Endocrine. (2016) 53:823–30. 10.1007/s12020-016-0949-y 27060006

[16] Lie B Leung E Leung PCK Auersperg N. Long-term growth and steroidogenic potential of human granulosa-lutein cells immoralized with SV40 large T antigen. Mol Cell Endocrinol. (1996) 120:169–76. 10.1016/0303-7207(96)03835-X 8832577

[17] Tu J Tian G Cheung H-H Wei W Lee T-L. Gas5 is an essential lncRNA regulator for self-renewal and pluripotency of mouse embryonic stem cells and induced pluripotent stem cells. Stem Cell Res Ther. (2018) 9:71. 10.1186/s13287-018-0813-5 29562912 PMC5863440

[18] Parikh A Lee C Joseph P Marchini S Baccarini A Kolev V. microRNA-181a has a critical role in ovarian cancer progression through the regulation of the epithelial-mesenchymal transition. Nat Commun. (2014) 5:2977. 10.1038/ncomms3977 24394555 PMC3896774

[19] Tu J Cheung H-H Lu G Chen Z Chan W-Y. MicroRNA-10a promotes granulosa cells tumor development via PTEN-AKT/Wnt regulatory axis. Cell Death Dis. (2018) 9:1076. 10.1038/s41419-018-1117-5 30348959 PMC6197200

[20] Jamieson S Fuller PJ. Molecular pathogenesis of granulosa cell tumors of the ovary. Endocr Rev. (2012) 33:109–44. 10.1210/er.2011-0014 22240241

[21] Shah SP Köbel M Senz J Morin RD Clarke BA Wiegand KC. Mutation of FOXL2 in granulosa-cell tumors of the ovary. N Engl J Med. (2009) 360:2719–29. 10.1056/NEJMoa0902542 19516027

[22] Schoof EM Lechman ER Dick JE. Global proteomics dataset of miR-126 overexpression in acute myeloid leukemia. Data Br. (2016) 9:57–61. 10.1016/j.dib.2016.07.035 27656662 PMC5021708

[23] Liu W Chen H Wong N Haynes W Baker CM Wang X. Pseudohypoxia induced by miR-126 deactivation promotes migration and therapeutic resistance in renal cell carcinoma. Cancer Lett. (2017) 394:65–75. 10.1016/j.canlet.2017.02.025 28257806 PMC5389460

[24] Wang J Zhou Y Fei X Chen X Zhu Z. Regulator of G-protein signaling 3 targeted by miR-126 correlates with poor prognosis in gastric cancer patients. Anticancer Drugs. (2017) 28:161–9. 10.1097/CAD.0000000000000446 27754994

[25] Guinn D Lehman A Fabian C Yu L Maddocks K Andritsos LA. The regulation of tumor-suppressive microRNA, miR-126, in chronic lymphocytic leukemia. Cancer Med. (2017) 6:778–87. 10.1002/cam4.996 28299881 PMC5387133

[26] Wang P Li Z Liu H Zhou D Fu A Zhang E. MicroRNA-126 increases chemosensitivity in drug-resistant gastric cancer cells by targeting EZH2. Biochem Biophys Res Commun. (2016) 479:91–6. 10.1016/j.bbrc.2016.09.040 27622325

[27] Wang C Zhou B Liu M Liu Y Gao R. miR-126-5p Restoration promotes cell apoptosis in cervical cancer by targeting Bcl2l2. Oncol Res. (2017) 25:463–70. 10.3727/096504016X14685034103879 28438233 PMC7841031

[28] Zhang W Zhou J Zhu X Yuan H. MiR-126 reverses drug resistance to TRAIL through inhibiting the expression of c-FLIP in cervical cancer. Gene. (2017) 627:420–7. 10.1016/j.gene.2017.06.055 28669929

[29] Grimolizzi F Monaco F Leoni F Bracci M Staffolani S Bersaglieri C. Exosomal miR-126 as a circulating biomarker in non-small-cell lung cancer regulating cancer progression. Sci Rep. (2017) 7:1–12. 10.1038/s41598-017-15475-6 29127370 PMC5681649

[30] Feng R Sah BK Beeharry MK Yuan F Su L Jin X Dysregulation of miR-126/Crk protein axis predicts poor prognosis in gastric cancer patients. Cancer Biomarkers. (2017) 1:1–9. 10.3233/CBM-170472 PMC13078259 29171987

[31] Jing B Ou Y Zhao L Xie Q Zhang Y. Experimental study on the prevention of liver cancer angiogenesis via miR-126. Eur Rev Med Pharmacol Sci. (2017) 21:5096–100. 10.26355/eurrev_201711_13825 29228448

[32] Luo J Zhu C Wang H Yu L Zhou J. MicroRNA-126 affects ovarian cancer cell differentiation and invasion by modulating expression of vascular endothelial growth factor. Oncol Lett. (2018) 15:5803–8. 10.3892/ol.2018.8025 29552211 PMC5840569

[33] Dong Y Fu C Guan H Zhang Z Zhou T Li B. Prognostic significance of miR-126 in various cancers: a meta-analysis. Onco Targets Ther. (2016) 9:2547–55. 10.2147/OTT.S103481 27217773 PMC4853159

[34] Xiang L Ou H Liu X Chen Z Li X Huang Y. Loss of tumor suppressor miR-126 contributes to the development of hepatitis B virus–related hepatocellular carcinoma metastasis through the upregulation of ADAM9. Tumor Biol. (2017) 39:101042831770912. 10.1177/1010428317709128 28639884

[35] Liu LY Wang W Zhao LY Guo B Yang J Zhao XG. miR-126 inhibits growth of SGC-7901 cells by synergistically targeting the oncogenes PI3KR2 and Crk, and the tumor suppressor PLK2. Int J Oncol. (2014) 45:1257–65. 10.3892/ijo.2014.2516 24969300

[36] Sun X Wang ZM Song Y Tai XUH Ji WY Gu H. MicroRNA-126 modulates the tumor microenvironment by targeting calmodulin-regulated spectrin-associated protein 1 (Camsap1). Int J Oncol. (2014) 44:1678–84. 10.3892/ijo.2014.2321 24603804

[37] Huang X Gschweng E Handel BV Cheng D Mikkola HKA Witte N. Regulated expression of microRNAs-126/126 ^*^ inhibits erythropoiesis from human embryonic stem cells. Blood. (2011) 117:2157–65. 10.1182/blood-2010-08-302711 21163928 PMC3062325

[38] Li H Chen S Liu J Guo X Xiang X Dong T. Long non-coding RNA PVT1-5 promotes cell proliferation by regulating miR-126/SLC7A5 axis in lung cancer. Biochem Biophys Res Commun. (2018) 495:2350–5. 10.1016/j.bbrc.2017.12.114 29277611

[39] Gong C Fang J Li G Liu H-H Liu Z-S. Effects of microRNA-126 on cell proliferation, apoptosis and tumor angiogenesis via the down-regulating ERK signaling pathway by targeting EGFL7 in hepatocellular carcinoma. Oncotarget. (2017) 8:52527–42. 10.18632/oncotarget.17283 28881749 PMC5581048

[40] Hu M Ma C Wang X Ye C Chen L Wang J. MicroRNA-126 inhibits tumor proliferation and angiogenesis of hepatocellular carcinoma by down-regulating EGFL7 expression. Oncotarget. (2016) 7: 66922–34. 10.18632/oncotarget.11877 27611944 PMC5341847

[41] Zhu J Li H Ma J Huang H Qin J Li Y. PTPN9 promotes cell proliferation and invasion in Eca109 cells and is negatively regulated by microRNA-126. Oncol Lett. (2017) 14:1419–26. 10.3892/ol.2017.6315 28789358 PMC5529898

[42] Wang J Zhou Y Fei X Chen X Yan J Liu B. ADAM9 functions as a promoter of gastric cancer growth which is negatively and post-Transcriptionally regulated by miR-126. Oncol Rep. (2017) 37:2033–40. 10.3892/or.2017.5460 28260063

[43] Boerboom D Paquet M Hsieh M Liu J Jamin SP Behringer RR Misregulated Wnt/b-catenin signaling leads to ovarian granulosa cell tumor development. Cancer Res. (2005) 65:9206–15. 10.1158/0008-5472.CAN-05-1024 16230381

[44] Laguë M-N Paquet M Fan H-Y Kaartinen MJ Chu S Jamin SP. Synergistic effects of Pten loss and WNT/CTNNB1 signaling pathway activation in ovarian granulosa cell tumor development and progression. Carcinogenesis. (2008) 29:2062–72. 10.1093/carcin/bgn186 18687666 PMC2577137

