# Supplementary material for: microRNA-126 Is a Tumor Suppressor of Granulosa Cell Tumor Mediated by Its Host Gene EGFL7
Source: Front Oncol. 2019 Jun 11;9:486. doi: 10.3389/fonc.2019.00486 (PMC6579899; doi:10.3389/fonc.2019.00486)
Supplement: Supplementary file 1 [file Image_1.pdf]

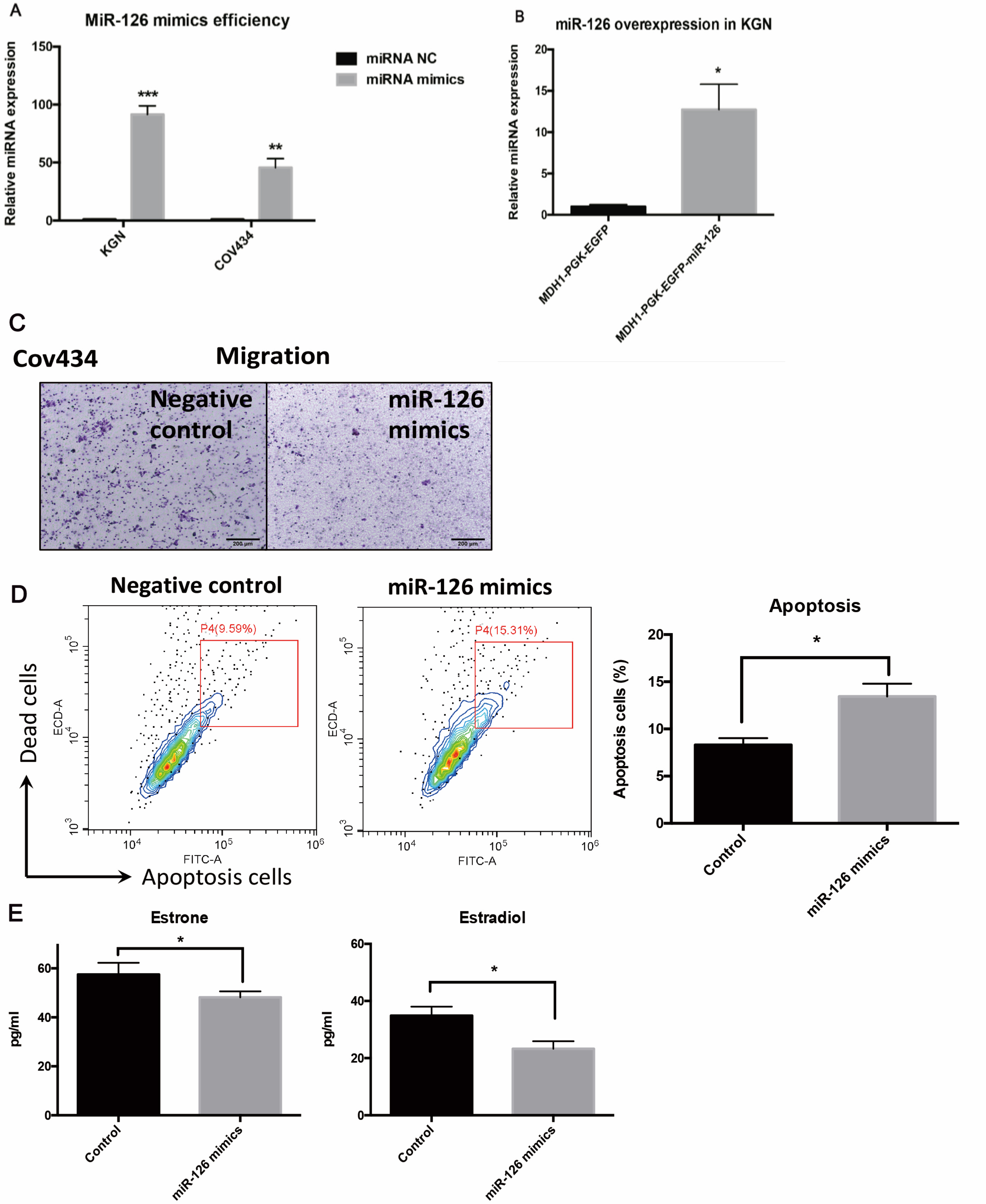

Supplementary figure 1. (A) The ovexpression efficiency of miR-126 mimics in KGN and Cov434 cells. (B) The overexpresison efficiency of miR-126 plasmid in KGN cells. (C) miR-126 repressed migration of Cov434 cells. (D) miR-126 induced apoptosis of Cov434 cells. (E) miR-126 inhibited production of estrone and estradiol. *P<0.05, **P<0.01, ***P<0.001
