# Supplementary material for: microRNA-126 Is a Tumor Suppressor of Granulosa Cell Tumor Mediated by Its Host Gene EGFL7
Source: Front Oncol. 2019 Jun 11;9:486. doi: 10.3389/fonc.2019.00486 (PMC6579899; doi:10.3389/fonc.2019.00486)
Supplement: Supplementary file 2 [file Image_2.pdf]

A

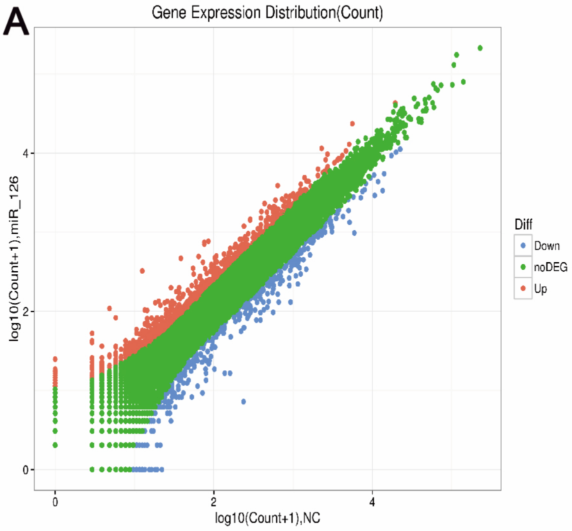

Supplementary figure 2. (A) The up-regulated and down-regulated genes in miR-126 overexpressing KGN cells. (B) Pathway enrichment by using all down-regulated gene from DAVID. (C) Pathway enrichment by using all up-regulated gene from DAVID.

B • Pathway enrichment by using all down-regulated genes

Sublist	Category	Term	RT	Genes	Count	%	P-Value	Benjamini
	KEGG_PATHWAY	Melanoma	RT		13	3.4	4.9E-9	1.0E-6
	KEGG_PATHWAY	Glioma	RT		11	2.9	2.5E-7	2.7E-5
	KEGG_PATHWAY	Rap1 signaling pathway	RT		18	4.8	2.6E-7	1.9E-5
	KEGG_PATHWAY	Pathways in cancer	RT		24	6.4	6.6E-7	3.5E-5
	KEGG_PATHWAY	Proteoglycans in cancer	RT		17	4.5	7.2E-7	3.0E-5
	KEGG_PATHWAY	Endometrial cancer	RT		9	2.4	4.2E-6	1.5E-4
	KEGG_PATHWAY	Prostate cancer	RT		11	2.9	4.4E-6	1.4E-4
	KEGG_PATHWAY	Non-small cell lung cancer	RT		9	2.4	7.5E-6	2.0E-4
	KEGG_PATHWAY	Ras signaling pathway	RT		15	4.0	6.8E-5	1.6E-3
	KEGG_PATHWAY	TNF signaling pathway	RT		10	2.7	1.4E-4	3.0E-3
	KEGG_PATHWAY	Central carbon metabolism in cancer	RT		8	2.1	1.6E-4	3.2E-3
	KEGG_PATHWAY	Focal adhesion	RT		13	3.4	4.1E-4	7.2E-3
	KEGG_PATHWAY	Estrogen signaling pathway	RT		9	2.4	4.6E-4	7.6E-3
	KEGG_PATHWAY	Bladder cancer	RT		6	1.6	8.9E-4	1.3E-2
	KEGG_PATHWAY	Colorectal cancer	RT		7	1.9	9.4E-4	1.3E-2
	KEGG_PATHWAY	ErbB signaling pathway	RT		8	2.1	1.1E-3	1.4E-2
	KEGG_PATHWAY	Thyroid hormone signaling pathway	RT		9	2.4	1.2E-3	1.5E-2
	KEGG_PATHWAY	Pancreatic cancer	RT		7	1.9	1.2E-3	1.4E-2
	KEGG_PATHWAY	Sphingolipid signaling pathway	RT		9	2.4	1.6E-3	1.8E-2
	KEGG_PATHWAY	PI3K-Akt signaling pathway	RT		16	4.2	1.7E-3	1.8E-2

C • Pathway enrichment by using all up-regulated genes

Sublist	Category	Term	RT	Genes	Count	%	P-Value	Benjamini
	KEGG_PATHWAY	Lysosome	RT		12	1.5	3.1E-3	5.0E-1
	KEGG_PATHWAY	Rheumatoid arthritis	RT		9	1.1	1.1E-2	7.1E-1
	KEGG_PATHWAY	Transcriptional misregulation in cancer	RT		13	1.6	1.4E-2	6.4E-1
	KEGG_PATHWAY	Malaria	RT		6	0.8	2.7E-2	7.8E-1
	KEGG_PATHWAY	HTLV-I infection	RT		16	2.0	3.3E-2	7.8E-1
	KEGG_PATHWAY	Viral myocarditis	RT		6	0.8	4.7E-2	8.3E-1
	KEGG_PATHWAY	mTOR signaling pathway	RT		6	0.8	5.0E-2	8.1E-1
	KEGG_PATHWAY	Hypertrophic cardiomyopathy (HCM)	RT		7	0.9	5.3E-2	7.8E-1
	KEGG_PATHWAY	Cell adhesion molecules (CAMs)	RT		10	1.3	5.8E-2	7.8E-1
	KEGG_PATHWAY	Glycosphingolipid biosynthesis - lacto and neolacto series	RT		4	0.5	6.0E-2	7.5E-1
	KEGG_PATHWAY	Ether lipid metabolism	RT		5	0.6	7.0E-2	7.7E-1
	KEGG_PATHWAY	Renin secretion	RT		6	0.8	7.1E-2	7.5E-1
	KEGG_PATHWAY	Systemic lupus erythematosus	RT		9	1.1	9.4E-2	8.2E-1
	KEGG_PATHWAY	FoxO signaling pathway	RT		9	1.1	9.4E-2	8.2E-1
