# Supplementary material for: microRNA-126 Is a Tumor Suppressor of Granulosa Cell Tumor Mediated by Its Host Gene EGFL7
Source: Front Oncol. 2019 Jun 11;9:486. doi: 10.3389/fonc.2019.00486 (PMC6579899; doi:10.3389/fonc.2019.00486)
Supplement: Supplementary file 3 [file Image_3.pdf]

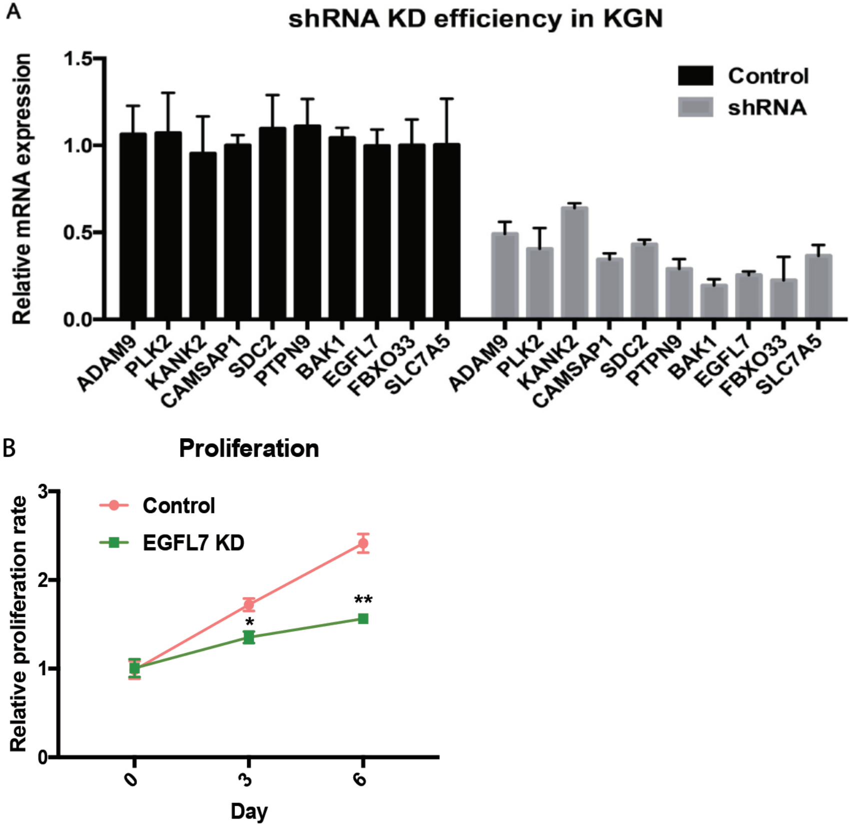

Supplementary figure 3. (A) The knockdown efficiency of all shRNAs in KGN cells. (B) The effects of EGFL7 KD on proliferation of KGN cells. *P<0.05, **P<0.01
